# Assessing the Views of Professionals, Patients, and Care Partners Concerning the Use of Computer Tools in Memory Clinics: International Survey Study

**DOI:** 10.2196/31053

**Published:** 2021-12-03

**Authors:** Aniek M van Gils, Leonie NC Visser, Heleen MA Hendriksen, Jean Georges, Majon Muller, Femke H Bouwman, Wiesje M van der Flier, Hanneke FM Rhodius-Meester

**Affiliations:** 1 Department of Neurology, Alzheimer Center Amsterdam, Amsterdam Neuroscience Amsterdam UMC, Location VUmc Amsterdam Netherlands; 2 Department of Neurobiology, Care Sciences and Society, Division of Clinical Geriatrics Center for Alzheimer Research Karolinska Institutet Stockholm Sweden; 3 Alzheimer Europe Luxembourg Luxembourg; 4 Department of Internal Medicine, Geriatric Medicine Section, Amsterdam Cardiovascular Sciences Institute Amsterdam UMC, Location VUmc Amsterdam Netherlands; 5 Department of Epidemiology and Data Science Amsterdam UMC, Location VUmc Amsterdam Netherlands

**Keywords:** artificial intelligence, clinical decision support systems, dementia, diagnostic testing, diagnosis, prognosis, communication

## Abstract

**Background:**

Computer tools based on artificial intelligence could aid clinicians in memory clinics in several ways, such as by supporting diagnostic decision-making, web-based cognitive testing, and the communication of diagnosis and prognosis.

**Objective:**

This study aims to identify the preferences as well as the main barriers and facilitators related to using computer tools in memory clinics for all end users, that is, clinicians, patients, and care partners.

**Methods:**

Between July and October 2020, we sent out invitations to a web-based survey to clinicians using the European Alzheimer’s Disease Centers network and the Dutch Memory Clinic network, and 109 clinicians participated (mean age 45 years, SD 10; 53/109, 48.6% female). A second survey was created for patients and care partners. They were invited via Alzheimer Europe, Alzheimer’s Society United Kingdom, Amsterdam Dementia Cohort, and Amsterdam Aging Cohort. A total of 50 patients with subjective cognitive decline, mild cognitive impairment, or dementia (mean age 73 years, SD 8; 17/34, 34% female) and 46 care partners (mean age 65 years, SD 12; 25/54, 54% female) participated in this survey.

**Results:**

Most clinicians reported a willingness to use diagnostic (88/109, 80.7%) and prognostic (83/109, 76.1%) computer tools. User-friendliness (71/109, 65.1%); Likert scale mean 4.5, SD 0.7), and increasing diagnostic accuracy (76/109, 69.7%; mean 4.3, SD 0.7) were reported as the main factors stimulating the adoption of a tool. Tools should also save time and provide clear information on reliability and validity. Inadequate integration with electronic patient records (46/109, 42.2%; mean 3.8, SD 1.0) and fear of losing important clinical information (48/109, 44%; mean 3.7, SD 1.2) were most frequently indicated as barriers. Patients and care partners were equally positive about the use of computer tools by clinicians, both for diagnosis (69/96, 72%) and prognosis (73/96, 76%). In addition, most of them thought favorably regarding the possibility of using the tools themselves.

**Conclusions:**

This study showed that computer tools in memory clinics are positively valued by most end users. For further development and implementation, it is essential to overcome the technical and practical barriers of a tool while paying utmost attention to its reliability and validity.

## Introduction

### Background

Dementia is a major health problem worldwide, with its prevalence expected to rise to 75 million patients in 2050 [1]. A timely and accurate diagnosis is essential for providing adequate care and appropriate treatment [2]. Diagnosing Alzheimer disease dementia or another type of dementia can be challenging, as clinical presentations overlap and multiple pathologies often co-occur [3,4]. Furthermore, increasing biomarker availability creates the possibility of diagnosing the early stages of Alzheimer disease before the onset of dementia and paves the way for individual dementia risk estimation [5,6].

With the availability of many different diagnostic tests, clinicians have the difficult task of combining and interpreting all the test results to come to an accurate diagnosis and prognosis [7-10] and communicating these results to patients and care partners [5,11,12]. Despite the increasing number of diagnostic tools, uncertainty in the diagnosis and prognosis of dementia remains, complicating the process of clearly explaining the test results [13]. Recent research has shown that most patients and care partners greatly value precise information on diagnosis and prognosis [12,14]. However, clinicians are often reluctant to address these topics during consultations, leaving these informational needs unmet [12,15].

Currently, artificial intelligence solutions are rapidly being developed and can aid clinicians in addressing these challenges in several ways. Artificial intelligence–based computer tools for dementia diagnosis and prognosis have demonstrated diagnostic accuracy equal to that of clinicians’ performance. These tools support individual risk estimation and increase clinicians’ confidence in diagnosis and prognosis [16-19]. Web-based cognitive test tools have shown promising results, enabling cost-effective testing [20-22]. From other medical fields, such as oncology, we know that computer-based tools can also support the communication process, for example, by engaging patients and their families more actively in the diagnostic decisions or by supporting clinicians in the clear communication of results [23,24]. To date, the actual implementation of such computer tools in memory clinic practice has been limited [25,26].

### Barriers and Facilitators

Several barriers to the acceptance and implementation of tools have been identified in different health care areas. The main concerns regarding computer tools are related to the physician–patient relationship: the fear of interfering with this relationship when using a tool and affecting patient communication. Furthermore, clinicians fear the disturbance of clinical work and the loss of clinical autonomy when using a tool. In addition, a time-consuming tool, a tool that does not fit into the workflow, complexity of a tool, and computer literacy have been frequently mentioned as barriers. On the other hand, good training before the use of a tool, user-friendliness, relevancy, transparency, and reliability are stimulating factors in the use of a computer tool in clinical practice [27-33].

It is not known if the same barriers and facilitators apply to computer tools in memory clinics. The nature of the patient population—older adults with cognitive decline—and the vast number of diagnostic tests involved in the diagnostic process might lead to a different set of relevant barriers and facilitators. In addition, patients’ and care partners’ opinions regarding the use of computer tools by their clinicians might be a potential barrier to or facilitator of clinicians’ use of a tool.

### Objective

Therefore, this study aims to understand preferences and identify the main barriers to and facilitators of using computer tools in the dementia workup from the perspectives of clinicians, patients, and care partners, that is, the end users.

## Methods

### Design

We conducted 2 surveys, 1 for clinicians and 1 directed at patients and care partners, both in the fall of 2020. In addition, to aid the interpretation of the survey results, we conducted an interactive panel session with clinicians. The study was approved by the medical ethical committee of the Amsterdam University Medical Center, Vrije Universiteit Medical Center, Amsterdam. All participants provided digitally informed consent.

### Survey for Clinicians

#### Participants

Between July and October 2020, we invited clinicians from memory clinics in Europe via the European Alzheimer’s Disease Consortium and the Dutch Memory Clinic network (Nederlands Geheugenpoli Netwerk [NGN]) through emails that contained a link to participate in the web-based survey. Furthermore, we invited all participants during the annual NGN conference to share their thoughts in our web-based interactive panel session.

#### Survey

The survey was created in the web-based survey tool Survalyzer (Survalyzer AG) [34] and translated into Dutch and English. The survey was adaptive; that is, certain questions were only conditionally displayed based on responses to other items. Furthermore, participants could scroll through the survey to edit their answers. The survey comprised 3 parts. In the first part, we collected background information (eg, age, gender, profession, and specialization). In the second part, we used a funneled method to examine the current opinions on computer tools and identify the barriers and facilitators. First, we asked if the clinician would be willing to use computer tools in general, after which we asked them to explain their opinions in an open-ended question. Subsequently, we provided them with a list of barriers and facilitators compiled from barriers and facilitators known from the existing literature [27,28,32], and we asked them which factors would stimulate or discourage them from using a tool. Clinicians could complement this list with their own perspectives. We then asked them to rate the importance of these factors using the Likert scale (1=very unimportant, 2=unimportant, 3=neutral, 4=important, and 5=very important). Finally, we asked the participants how likely they were to use *diagnostic* and *prognostic* tools. In the third part, we explored clinicians’ opinions on additional computer tools, that is, web-based cognitive testing, communication support, and communication skills training.

#### Interactive Panel Session

During the NGN annual conference (held on the web on November 10, 2020), 2 authors (HFMRM and LNCV) presented the preliminary results of the survey. To help interpret these results, they asked all conference participants several in-depth questions using Mentimeter [35]. These questions were related to the importance of several factors that stimulate their trust in a tool; factors that would convince them of the usability, reliability, and validity of a tool; and the primary outcome measures of a tool.

### Survey for Patients and Care Partners

#### Participants

Between July and October 2020, we invited a mixed memory clinic population comprising patients with subjective memory complaints (subjective cognitive decline [SCD]), mild cognitive impairment, and dementia and care partners to participate in the web-based survey. To be included, patients had to be able to understand the questionnaire in Dutch or English. In this study, *care partner* refers either to an informal caregiver or a close relative or friend of the patient who provides either or both emotional and practical support. A general link to the survey was sent via a newsletter and social media to the members (patients and care partners) of Alzheimer Europe and directly to the members of the Alzheimer’s Society United Kingdom. Next, we invited both patients and care partners from the Amsterdam Dementia Cohort of the Alzheimer Center Amsterdam [36,37] and the Amsterdam Aging Cohort of the outpatient geriatric clinic of the Amsterdam University Medical Center (Amsterdam University Medical Centers) [38]. Patients and care partners were approached and informed by phone or email, and when they confirmed their participation, they were sent a personalized link to the web-based survey. The survey was adaptive to reduce the number of questions. Furthermore, participants could scroll through the survey to edit their answers.

#### Survey

A total of 2 versions of the survey were created, 1 directed at patients and 1 at care partners, both comprising 3 parts. In part 1, we collected background information regarding the participants (eg, age, gender, and diagnosis). In part 2, we asked for their opinion on clinicians’ use of (1) a computer tool that analyzes the results of the diagnostic tests (*diagnostic tool*), (2) a computer tool to help predict the course of their symptoms (*prognostic tool*), and (3) a tool to help communicate the test results in *day-to-day* language with the patient (*communication tool*). We adjusted the predefined list of barriers to and facilitators of using computer tools to the patient and care partner perspectives. We provided participants with this list and asked the extent (1=strongly disagree, 2=disagree, 3=neutral, 4=agree, and 5=strongly agree) to which the different items applied to them. In the last part, we asked their opinion on computer tools directed at patients and care partners, that is, web-based cognitive testing and tools that could support and empower them in their communication with the clinicians. The survey was piloted in a test panel of 3 patients (2 with SCD and 1 with dementia) and 1 care partner of a person with dementia.

### Analysis

Completion of the survey was enforced using mandatory questions. Proceeding with the survey was not possible when a question was unanswered. Furthermore, it was not possible to complete the survey more than once, as Survalyzer solely allowed unique visitors. Only completed surveys were analyzed. We analyzed participant characteristics and survey outcomes using descriptive statistics. Chi-square tests were used to compare answers between patients and care partners. For clinicians, we compared answers between groups based on age, sex, profession, and specialization. When using the 5-point Likert scale, the mean Likert scale scores were calculated per item. Frequencies were calculated for all the barriers and facilitators from the predefined list. We combined the frequencies with mean Likert scale scores to define the most important barriers and facilitators (eg, the item with the highest frequency combined with the highest mean Likert scale score was regarded as the most important). SPSS, version 22.0 (IBM Corporation) was used to analyze the quantitative data. *P* values <.05 were considered significant.

The answers to the open-ended questions were analyzed in MAXQDA software (VERBI Software) [39] using a process of deductive thematic content analysis [40,41]. A total of 2 authors, AMVG (physician) and HMAH (neuropsychologist), independently generated the initial thematic codes based on the existing literature and data. Subsequently, 1 author (AMVG) generated a thematic framework and used this framework to code all the given answers. The codes were then sorted into broad categories.

## Results

### Demographics

Sample descriptions have been presented in Table 1 for clinicians and Table 2 for patients and care partners. Clinicians were, on average, aged 45 (SD 11) years and had 16 (SD 13) years of experience. Most participating clinicians were medical specialists working in neurology (60/109, 55%) or internal or clinical geriatric medicine (33/109, 30.3%). Patients were in general older (mean age 73 years, SD 8) than care partners (mean age 65 years, SD 12), who were mostly a partner or spouse (33/46, 72%) or a granddaughter, daughter, grandson, and son (in-law; 12/46, 26%). Participating patients were most often diagnosed with SCD (21/50, 42%), whereas participating care partners were mostly those of patients with dementia (36/46, 78%).

**Table 1:** Sample demographics of clinicians participating in the web-based survey and interactive panel session (N=294).

Characteristics			Web-based survey (n=109)		Interactive panel session^a^ (n=184)
Age (years), mean (SD)			45 (11)		43 (11)
Sex (female), n (%)			53 (48.6)		98 (85.9)
**Cohort, n (%)**					
	European Alzheimer’s Disease Consortium	53 (48.6)		N/A^b^	
	Dutch Memory Clinic network	56 (51.4)		N/A	
**Profession, n (%)**					
	MD^c^, specialist	87 (79.8)		60 (54.5)	
	MD, specialist training or not in specialist training	12 (10.9)		1 (0.9)	
	Physician assistant, nurse specialist, or specialized nurse	3 (2.8)		23 (20.9)	
	Neuropsychologist or psychologist	6 (5.5)		16 (114.5)	
	Other	1 (0.9)		10 (9.1)	
Experience^d^ (years), mean (SD)			16 (13)		N/A
**Specialization^e^, n (%)**					
	Neurology	60 (50.4)		N/A	
	Clinical geriatric or internal medicine	33 (30.3)		N/A	
	Nursing home physician or general practitioner	2 (1.8)		N/A	
	Psychiatry	9 (8.3)		N/A	
	Other	9 (8.3)		N/A	
**Institution^f^, n (%)**					
	Academic or university hospital	68 (62.4)		N/A	
	Nonacademic teaching hospital	32 (29.4)		N/A	
	Nonteaching hospital	8 (7.3)		N/A	
	Mental health service	2 (1.8)		N/A	
	Other	3 (2.8)		N/A	

^a^Owing to the hybrid conference setting, not every description was available for all participants. For sex, n=114 participants replied to the question. For profession, n=110 participants replied to the question.

^b^N/A: not applicable.

^c^MD: medical doctor.

^d^Only applicable for medical specialists.

^e^Some clinicians had ≥1 specialization.

^f^Some clinicians worked in ≥1 institution.

**Table 2:** Sample demographics of patients and care partners participating in the web-based survey (N=96).

Characteristics		Patients^a^ (n=50)	Care partners (n=46)
Age (years), mean (SD)		73 (8)	65 (12)
Sex (female), n (%)		17 (34)	25 (54)
**Cohort, n (%)**			
	Alzheimer Europe or Alzheimer’s Society United Kingdom	2 (4)	14 (30)
	Amsterdam dementia cohort	25 (50)	27 (52)
	Amsterdam aging cohort	23 (46)	5 (18)
**Diagnosis^b^, n (%)**			
	SCD^c^	21 (42)	2 (4)
	MCI^d^	16 (32)	8 (17)
	Dementia	13 (26)	36 (78)
**Education^e^, n (%)**			
	Low	1 (2)	1 (2)
	Middle	22 (45)	16 (36)
	High	26 (53)	27 (61)

^a^Of these 50 patients, 20 (40%) completed the survey together with their care partner.

^b^For the care partners, the numbers represent the diagnosis of their loved ones.

^c^SCD: subjective cognitive decline.

^d^MCI: mild cognitive impairment.

^e^According to the Dutch Verhage scale (low 1-3; middle 5; high 6-7).

### Survey of Clinicians

#### Opinions on the Use of Computer Tools

In response to whether they would be willing to use computer tools in their daily clinical practice, 51.4% (56/109) of clinicians said they would probably use a diagnostic tool, and 29.4% (32/109) said they would certainly use a diagnostic tool. Furthermore, 7.3% (8/109) said they would be unlikely to use a tool, 11.9% (13/109) answered neutrally, and none of the clinicians reported that they did not want to use a tool. The results were similar for prognostic tools; of the 109 clinicians, 53 (48.6%) said they would probably use a prognostic tool, and 30 (27.6%) said they would certainly use a prognostic tool. Furthermore, 2.8% (3/109) were unlikely to use a prognostic tool, and 0.9% (1/109) would certainly not use a prognostic tool. The remaining participants responded as neutral (22/109, 20.2%). We found no differences in both diagnostic and prognostic tools based on age (*P*=.20 and *P*=.49, respectively), sex (*P*=.14 and *P*=.73, respectively), or profession (*P=*.61 and *P*=.98, respectively). We found that neurologists indicated more willingness to use prognostic tools (*P*=.04) than clinicians from other specializations.

Content analysis of clinicians’ explanations of their opinion on the use of computer tools resulted in 6 main topics: *support, clinical expertise, efficiency, accuracy, clinician–patient relationship*, and *care of the future*. Each of the topics has been described in Table 3, and illustrative quotes have been provided.

**Table 3:** Clinicians’ opinions on the use of computer tools in memory clinics.

Topics	Description		Quotes
	Facilitating factors	Hindering factors	
Support	Support to the diagnostic process from screening or prescreening to follow up, support data storage, support research purposes	Not applicable for specific patient populations	“[...] I would welcome a tool that would be implemented with the available clinical data and help reach a diagnosis (ie, considering the neurocognitive and neuroimaging data, in patients with such profile, expert diagnosis would be...with a probability of...%—which could be increased by the use of...biomarker).” [Male, 42 years, MD^b^, physician working in neurology]
Clinical expertise	Complementary to clinical expertise (eg, an aid for complex cases or with interpretation of test results) and contributory to evidence-based medicine [42]	Tools should not be a replacement for clinical expertise	“Computer tools and AI might be a way to have an evidence-based standard procedure in addition to my own long time clinical experience.” [Female, 59 years, MD, geriatrician]; “[...] I consider the clinical view as most important. A computer tool cannot (partly) replace this.” [Female, 38 years, MD, geriatrician]
Efficiency	The ability to standardize the diagnostic process, if easy to use, if connecting with electronic patient file, and time-efficiency	A tool not connected to the electronic patient file, information technology issues	“A quick and useful way to get practical answers on the workplace.” [Male, 62 years, MD, neurologist]; “For tools that are not implemented in the electronic patient file I foresee barriers in the implementation.” [Female, 45 years, MD, geriatrician]
Accuracy	Computer tools could help in making a more accurate diagnosis, providing additional objective information, and overcoming human errors	Tools might generate results of no use and fear of loss of important clinical information	“Sometimes we can be influenced by the patient we have in front of us. We can diagnose them too easily or consider them as (sub)normal because their general behavior makes us think so. A computer could be more objective than we are in some cases.” [Male, 26 years, MD, neurology resident]; “[...] I am afraid that there will be an outcome that is of no use for me, such as 64% chance of Alzheimer’s disease.” [Female, 38 years, MD, geriatrician]
Clinician–patient relationship	Improving patient communication	A tool might have a negative impact on the relationship between clinicians and patients	“[...] It facilitates the communication to the patient.” [Female, 49 years, MD, neurologist]; “Patients also come for attention and care, which they get less if we look at the screen more often.” [Male, 32 years, MD, physician working in neurology]
Care of the future	The use of tools is considered part of the care of the future	N/A^a^	“AI and big data are the future, they make the invisible visible [...].” [Male, 33 years, MD, internal (geriatric) medicine resident]

^a^N/A: not applicable.

^b^MD: medical doctor.

#### Barriers and Facilitators

**Figure 1:**
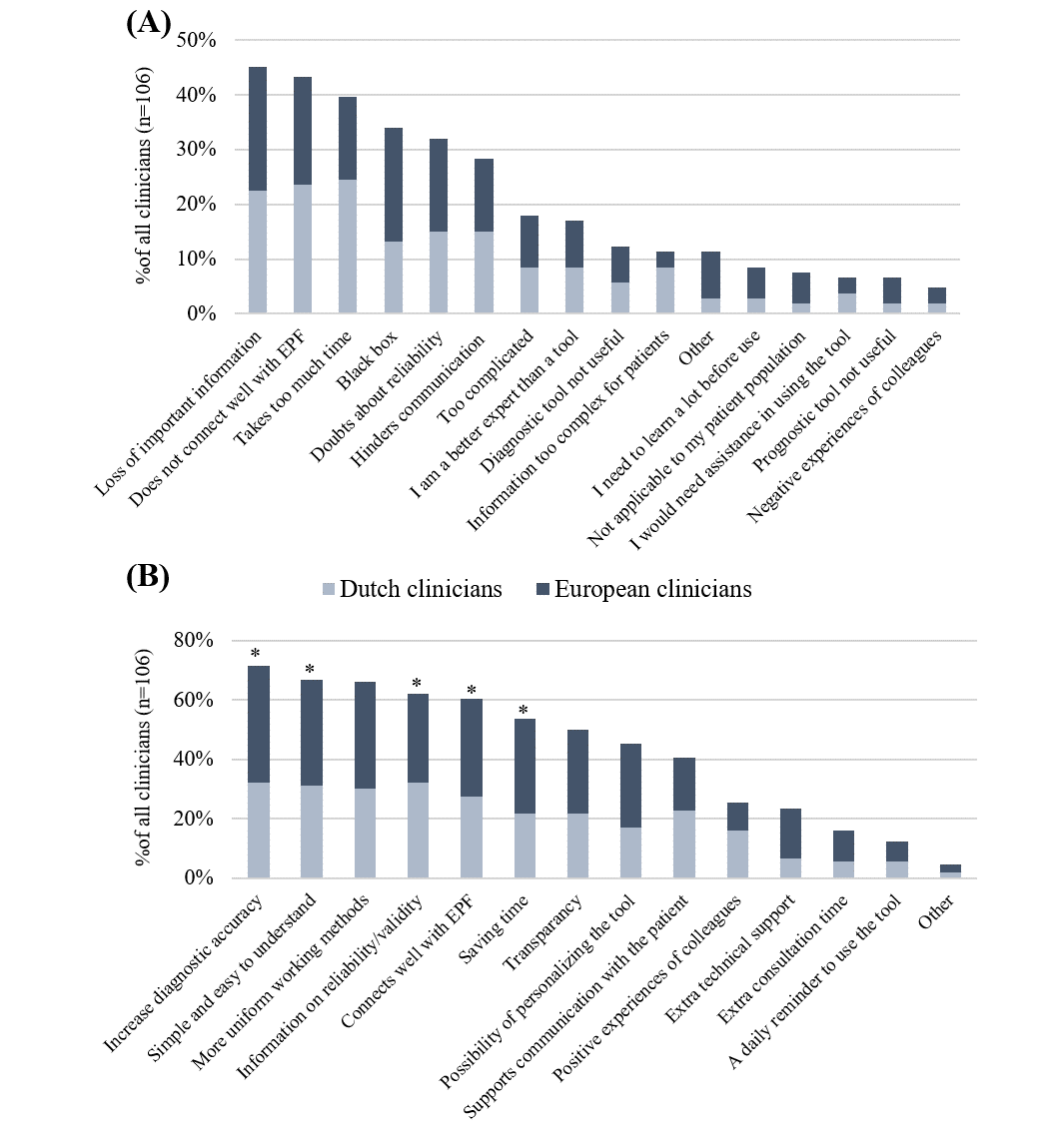
Frequencies of barriers to (A) and facilitators of (B) the use of computer tools in daily practice according to Dutch and European clinicians. EPF: electronic patient file. *Items rated with a mean Likert scale score of ≥4.

Clinicians’ selection of factors that would hinder the use of computer tools has been displayed in Figure 1A. None of the barriers were rated using a mean Likert scale score ≥4. Figure 1B shows which factors would stimulate the use of computer tools; items marked with an asterisk were rated with a mean Likert scale score of ≥4. The most important barriers were (1) a tool not being well connected with the electronic patient file (47/109, 43.2% of clinicians), with a mean Likert scale score of 3.8 (SD 1.0), and (2) important information, such as observations, cannot be added to a tool (49/109, 44.9% of clinicians), with a mean Likert scale score of 3.7 (SD 1.2). The most important facilitators were (1) user-friendliness (73/109, 66.9% of clinicians; mean 4.5, SD 0.7), and (2) increasing diagnostic accuracy (78/109, 71.6% of clinicians; mean 4.3, SD 0.7).

#### Additional Tools for Memory Clinics

Many clinicians (48/109, 44%) reported a willingness to use both web-based communication tools and skills training. Over half of the clinicians (62/109, 56.9%) indicated that they would like to test the patient’s cognition via the computer at home before the appointment. Frequently selected reasons for web-based cognitive testing were triage (62/109, 56.9%) and shortening of the test battery at the clinic (37/109, 33.4%). Not being able to observe the patient during testing was mentioned by 89% (42/47) of the clinicians who would not want to test the patient’s cognition on the web.

#### Interactive Panel Session

Both transparencies about the objectives of the tool provider (Likert scale score mean 4.3, SD 1.1) and honesty about the possibilities and limitations of a tool (Likert scale scores mean 4.4, SD 0.9) were considered important to strengthen clinicians’ trust in a tool. The most important aspects of convincing clinicians of usability, reliability, and validity of a tool were the explicit provision of information regarding the tool (mean 4.3, SD 0.9), scientific publications on underlying models (mean 4.3, SD 0.9), obtaining hands-on experience with the tool (mean 4.2, SD 0.6), and a randomized controlled trial (RCT) to test the effectiveness of the tool in clinical practice (mean 4.0, SD 0.8). Diagnostic accuracy (31/100, 31%) and patient-related outcome measures (38/100, 38%), such as quality of life, were most frequently selected as the ideal primary outcome measures of such an RCT.

### Survey of Patients and Care Partners

#### Tools Used by Clinicians

The results of patients’ and care partners’ opinions on diagnostic, prognostic, and communication tools have been presented in Table 4. Most patients and care partners were positive regarding their clinician using these tools. No differences were found between patients and care partners (diagnostic tools, *P*=.36; prognostic tools, *P=*.36; communication tools, *P*=.63) or different syndrome diagnoses (diagnostic tools, *P*=.64; prognostic tools, *P=*.69; communication tools, *P*=.92).

**Figure 2:**
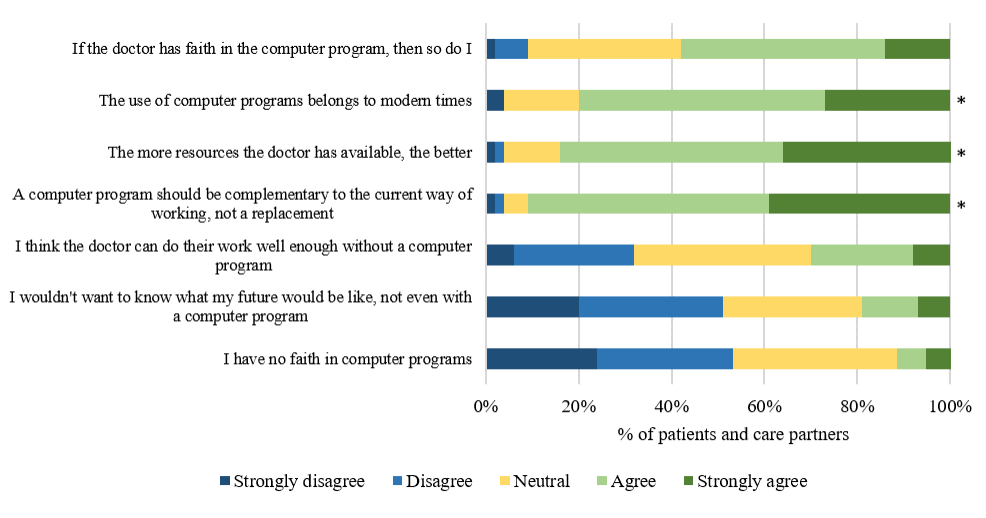
Agreement of patients and care partners on several statements regarding computer tools on a 5-point Likert scale. * Items rated with a mean Likert scale score of ≥4.

Figure 2 shows an overview of the responses of patients and care partners to several statements regarding the use of computer tools by clinicians. Items marked with an asterisk are items rated with a mean Likert scale score ≥4. We found no differences between the responses of patients and those of the care partners (*P* values in order of appearance of topics from top to down in the figure: *P*=.75, *P*=.62, *P*=.70, *P*=.55, *P*=.78, *P*=.21, and *P=*.60).

**Table 4:** Opinion of patients and care partners on clinicians’ use of diagnostic, prognostic, and communication tools, illustrated with quotes (N=96).

Opinion		Patients (n=50), n (%)	Care partners (n=46), n (%)	Quotes
**Diagnostic tool**				
	I think that is a good thing	38 (76)	31 (67)	“The more information, the better. As long as the computer program is in addition to the doctor’s expertise and not a replacement, I think it would be a good idea.” [Female 60 years, care partner]
	I would not want that	4 (8)	4 (9)	“I think face to face contact between the doctor and the patient is essential.” [Female 74 years, care partner]
	I do not know or no opinion	8 (16)	11 (24)	“Depends on how good the program is.” [Male 76 years, patient, dementia]
**Prognostic tool**				
	I think that is a good thing	41 (82)	32 (70)	“There is nothing against the use of a computer in predicting the disease process. It remains an aid to the physician. [...] He/she should remain leading.” [Male 78 years, patient, SCD^a^]
	I would not want that	4 (8)	6 (13)	“I want to know so I can plan ahead. However, with the variation in the progression rate, I don’t see how this could be sufficiently accurate. If not accurate, I would not want it.” [Female, 61 years, patient, dementia]
	I do not know or no opinion	5 (10)	8 (17)	“My husband approves [the use of a prognostic tool], me as his wife, do not know if I would like it. What if the prediction is somber! We would instantly be depressed.” [Female (age unknown), care partner]
**Communication tool**				
	I think that is a good thing	39 (78)	38 (83)	—^b^
	I would not want that	3 (6)	1 (2)	—
	I do not know or no opinion	8 (16)	7 (15)	—

^a^SCD: subjective cognitive decline.

^b^No quotes available.

#### Tools Directed at Patients and Care Partners

Most patients (35/50, 70%) and care partners (26/46, 57%) expressed a preference for a list of example questions to select questions they wanted to ask their clinician. Furthermore, 42% (21/50) of patients and 50% (23/46) of care partners said they would prepare their visit to the memory clinic by watching informational videos. A smaller but considerable proportion of care partners (11/46, 24%) indicated that they would use a web-based communication tool to practice their communication skills, and some (5/50, 10%) patients reported wanting to do the same. A small proportion of patients (4/50, 8%) and care partners (8/46, 17%) did not want to use any web-based communication tools.

Most patients (35/50, 70%) and care partners (28/46, 61%) were positive regarding web-based cognitive testing at home. Reasons for not wanting to perform cognitive testing at home differed between patients and care partners (*P*=.01). The most frequently selected reason for patients was their preference for personal contact with the clinician (18/26; 69%). The most selected reason (5/12, 42%) for care partners was that web-based cognitive testing was too difficult to perform for their loved ones.

## Discussion

### Principal Findings

This study showed that most clinicians, patients, and care partners were supportive of the use of computer tools in memory clinics. This holds true for diagnostic and prognostic tools, tools that support communication, and web-based cognitive testing. Despite acknowledging their potential barriers, the general attitude of clinicians toward these tools was positive. The facilitating factors were mainly practical (tools should be user-friendly) and technical (connection with electronic patient files) and that tools should increase diagnostic accuracy. The identified barriers mainly focused on doubts regarding reliability and validity, preservation of clinical autonomy, and fear of losing important clinical information. Furthermore, the tools should be considered in addition to the current working methods and not as a replacement.

We hypothesized that the barriers to and facilitators of tools in memory clinics might differ from those identified in other health care areas because of the nature of the patient population (older adults with cognitive decline) and the large number of diagnostic tests used in the clinical workup of dementia. However, the barriers and facilitators we found in this survey study largely corresponded to the existing literature on barriers to and facilitators of the use of computer tools in other medical fields [27-33]. Furthermore, we found that most of the given answers to the open-ended question were in accordance with our predefined list, which was based on studies on applying computer tools in other health care areas [27,28,32]. In addition, it is conceivable that patients’ and their care partners’ (negative) opinions regarding the tools were a possible barrier to clinicians using a tool. In this study, we showed that patients, despite their age and (potential) cognitive decline, are mainly positive regarding the use of computer tools. Most of them embrace the possibility of using a tool themselves, and their care partners share this opinion. A computer tool must support rather than replace clinicians, who would then view it as an aid appropriate to modern times.

### Increase Acceptance of Tools

We found the overall attitude among clinicians toward tools to be highly positive. Nevertheless, none of the available tools are regularly used in daily practice, and it seems there is a major information gap and educational need to make clinicians understand the possibilities of such computer tools [43]. The results from our survey provide direction for the way to increase the acceptance of computer tools in memory clinic practice. First, clinicians indicated that an RCT on the efficiency in clinical practice would boost their confidence in the reliability of computer tools. RCTs are considered important and robust methods for assessing the impact of a tool [44,45]. However, no RCT on the application of computer tools has yet been performed in memory clinics. Thus, our findings suggest a need for RCTs with diagnostic accuracy and patient-related outcome measures as primary outcome measures.

Second, computer tools could contribute to evidence-based medicine (EBM) [42]. EBM concerns medical practice based on the best available evidence, clinical experience, and patient preferences [42]. Within the concept of EBM, clinical experience is highly essential and should not be replaced by a tool. Computer tools could support EBM by making the best available evidence more accessible to clinicians, and computer tools could clarify patient preferences. Acknowledging computer tools as a part of EBM might lead to clinicians viewing these tools as an aid complementary to their own clinical experience rather than a threat to their clinical autonomy [27,29,32,46]. In addition, there must be scientific publications regarding the underlying models and the transparent provision of information regarding a tool’s reliability to increase clinicians’ trust in the tools [28,32,47].

Then, clinicians’ confidence in the tools might be strengthened if the tools are under the jurisdiction of a regulatory body to authorize and supervise the quality. To date, there are no formal regulatory standards for tools used to support clinicians in decision-making [28]. When developers claim that their software has a medical purpose, it becomes a medical device, and manufacturers themselves have to proclaim that their device meets the safety and performance requirements. The new European Medical Devices Regulation implemented in May 2021 makes manufacturers adhere to more strict guidelines for ensuring the safety of their products, including assessment of their device by a notified body. Involving an independent notified body in the approval of a tool might be the first step toward improving its transparency and acceptance. Eventually, as a next step, the use of software should be included in the guidelines of professional associations [26].

The attitude toward tools is one of the key characteristics of eventual acceptance [30,32]. Several user acceptance models have been proposed to further encourage the acceptance of tools in medical practices [32]. In this context, we would like to address the user acceptance and system adaptation design model [32]. This model aims to include end users as the central point in the design process of a computer tool. In this model, user expectations and needs need to be thoroughly understood before developing a tool. The development of a tool is an iterative process, and end users should be continuously involved throughout the development process. In congruence with this user acceptance model, we took the first step toward accepting computer tools by identifying the barriers to and facilitators of computer tools according to the end users in memory clinics. On the basis of the user acceptance and system adaptation design model, the next step in the iterative process would be the evaluation of usability. Then, a pilot study would be needed to evaluate user acceptance. The results of each step should direct improvements in the tool and be evaluated in the next step. However, it should be mentioned that in this study, we asked participants about the barriers to and facilitators of computer tools in general. Therefore, the barriers and facilitators found in this study should be considered a starting point. When implementing specific tools, further exploration of user expectations and needs for those specific tools might be necessary.

### Strengths and Limitations

One of the strengths of this study is that we involved all the end users of computer tools in memory clinics, that is, clinicians, patients, and care partners. Including patients and care partners originating from Europe and a heterogeneous population of clinicians contributed to the generalizability of the results. Furthermore, we used a funneled method in which we started the survey with open-ended questions and worked toward closed questions. By doing so, we actively asked and stimulated clinicians’ own input.

Nevertheless, our study has several limitations. First, we distributed the survey via a web-based link, which might have caused selection bias by only involving people with sufficient digital skills who might have had a more positive approach toward computer tools. We tried to minimalize this risk for patients and care partners by including participants from both geriatric and neurology departments with different cognitive impairment stages. Furthermore, participants’ ages ranged from relatively young to older patients, who might have had less experience with digital tools. Second, we have no data available on the origin of the European participants, which might have led to an uneven distribution of participants among countries. Nonetheless, the international character of this study contributes to the generalizability of the results. Third, there might be a risk of response bias; people who are less inclined to use computer tools may not have responded. We could not estimate the response rates as the link to the survey was spread among an unknown number of people. Nonetheless, we gained insight into the important barriers and facilitators based on inquiry among large samples of the most important stakeholders.

### Conclusions

In conclusion, this study shows broad support for the use of computer tools in memory clinic practices by clinicians, patients, and care partners. To stimulate the implementation of tools in daily memory practice, the tools should overcome several technical and practical barriers. Moreover, clinicians have to be convinced regarding the reliability and validity of the tool. By identifying the potential barriers and facilitators, we have paved the way for further development and implementation of the tools. Our results provide an important step in the iterative process of developing computer tools for memory clinics in cocreation with end users.

## Abbreviations

EBM: evidence-based medicine
NGN: Nederlands Geheugenpoli Netwerk
RCT: randomized controlled trial
SCD: subjective cognitive decline
